# AGING-IN-PLACE TECHNOLOGY: PROGRAMMING TO ADDRESS SOCIAL CONNECTEDNESS AND MENTAL HEALTH

**DOI:** 10.1093/geroni/igad104.1351

**Published:** 2023-12-21

**Authors:** Keith Morgen, Peter Jacob

**Affiliations:** Centenary University, Hackettstown, New Jersey, United States; Jewish Family Service of Central New Jersey, Elizabeth, New Jersey, United States

## Abstract

According to the National Academies of Sciences, Engineering, and Medicine, the drastic increase of older adult social isolation from the pandemic has contributed to premature death, dementia, anxiety, and suicide. Jewish Family Service of Central New Jersey (JFSCNJ) clients corroborated this phenomenon with 65% meeting the diagnostic criteria for moderate to severe older adult depression or anxiety in 2020. In 2022, JFSCNJ established the Fig Tree Virtual Senior Center, a dynamic program that focused on older adults as they readjust to a post-pandemic world. By addressing social isolation through a Person-Centered Trauma Informed and biopsychosocial approaches, various wellness programs were provided in-home via the use of virtual video programming. Sixteen older adults (age range 68-95 years-old) were assessed on anxiety and depressive symptoms and social connectedness both prior to and six months following the start of the video programming intervention. Paired samples t-testing found a significant reduction in self-reported anxiety (p=.01) and depressive (p=.05) symptoms after video programming engagement at six-months follow-up. Path analysis using bootstrapping with bias corrected confidence intervals found amount of video programming with a positive and significant indirect effect on both increased social connectedness and improved well-being. Implications for homebound older adult social engagement and well-being programming will be discussed.

